# Reactive Attachment Disorder in the General Population: A Hidden ESSENCE Disorder

**DOI:** 10.1155/2013/818157

**Published:** 2013-04-18

**Authors:** Rachel Pritchett, Jennifer Pritchett, Emma Marshall, Claire Davidson, Helen Minnis

**Affiliations:** ^1^Academic Unit of Mental Health & Wellbeing, University of Glasgow, Caledonia House, Royal Hospital for Sick Children, Glasgow G3 8SJ, UK; ^2^Psychological Services, North Lanarkshire Council, St Brendan's Primary School, 45 Barons Road, Motherwell ML1 2NB, UK; ^3^Young People In Mind Service, Vale of Leven Hospital, Alexandria G83 0UA, UK

## Abstract

Reactive attachment disorder (RAD) is a severe disorder of social functioning. Previous research has shown that children with RAD may have poor cognitive and language abilities; however, findings mainly come from biased, institutionalised samples. This paper describes the characteristics of all children who were given a suspected or likely diagnosis of reactive attachment disorder in an epidemiological study of approximately 1,600 children investigating the prevalence of RAD in the general population. We found that children with RAD are more likely to have multiple comorbidities with other disorders, lower IQs than population norms, more disorganised attachment, more problem behaviours, and poorer social skills than would be found in the general population and therefore have a complex presentation than can be described as ESSENCE. We discuss the clinical and educational implications.

## 1. Introduction

Reactive attachment disorder (RAD) is a severe disorder of social functioning. It has two subtypes: inhibited type, where the child will display wary, watchful, and hypervigilant behaviours and disinhibited type, where the child displays indiscriminately friendly behaviours, engages socially with strangers, and shows no need to remain near the safety of their primary caregiver [1]. It is thought that RAD is a result of severe maltreatment in early childhood, and there is research indicating that adopted children will be more likely diagnosed as having RAD than children raised by a biological parent [2].

In addition to the core features described above, there are numerous symptoms associated with RAD for example, Stinehart et al. [1] describe some potential early symptoms including failure to gain weight or feeding difficulties developing into unusual eating habits, lack of empathy, or impulse control which could lead to criminal behaviours and cruelty to animals as the child grows older. Both DSM and ICD state that core symptoms of indiscriminate friendliness or emotional withdrawal/hypervigilance need to be present before age 5. We would therefore describe RAD as having early symptomatic symptoms eliciting neurodevelopmental examination (ESSENCE).

There have been many studies which have examined cognitive and developmental characteristics of children with this disorder. Minnis et al. [3] compared children who had been referred by a clinician as they were suspected to have RAD with a general population comparison group. They found that children with RAD had significantly higher problem scores on both parent and teacher reports in the Strengths and Difficulties Questionnaire (SDQ) which covers symptoms of conduct problems, emotional problems (anxiety and depression), hyperactivity, and peer relations. They also had a lower receptive vocabulary than the comparison group, on the British Picture Vocabulary Scale (BPVS). A further study with these children showed that children with RAD demonstrate significant problems in social relatedness and pragmatic language skills with a degree of severity equivalent to children in an ASD comparison group [4]. 

Kocovska et al. [5] looked at the characteristics of adopted children with a history of severe maltreatment, who were indiscriminately friendly. They found that these children had a lower IQ than a comparison group, were more likely to have language problems and to have several comorbid psychiatric disorders. 

Some of the key research in this area comes from the Bucharest Intervention Studies. Zeanah et al. [6] have published widely on their randomised controlled trial of foster placement as an alternative to institutionalisation in abandoned infants and toddlers in Romania. They have found high levels of Reactive Attachment Disorder symptoms in the sample and associations between RAD and lower cognitive ability [7]. In addition, their results show adverse effects of poor institutional care on later language development [8], and found attachment security as an important mediator of the relationship between the quality of early caregiving and later psychopathology [9].

These important studies have shown that children with RAD may experience additional problems affecting both development and future outcomes. Gillberg [10] discussed the coexistence of disorders, concluding that the sharing of symptoms across disorders is the rule rather than the exception and argued that numerous childhood disorders such as autism spectrum disorder, attention deficit hyperactivity disorder, and reactive attachment disorder all share symptoms in the early stages which should be treated by a multidisciplinary team of specialists. RAD has not traditionally been considered to be a neurodevelopmental disorder, as it is thought to be caused by maltreatment, but it may be that maltreatment in early life can set in train developmental trajectories that are shared by other ESSENCE disorders.

Because all of the previous research has been conducted in clinical or otherwise select samples, such as children from institutions, we were keen to explore the difficulties which children with RAD encounter while living in the general population. Minnis and colleagues [11] conducted the first epidemiological study focussing on the prevalence of RAD in the general population and found a prevalence of 1.4%. With such a high prevalence of RAD in the general population, it is imperative to understand the additional needs of these children. This study describes the characteristics of the children identified as having RAD in this sample. We were interested to explore whether children with RAD in the general population also have complex, overlapping problems i.e. are they an example of children with ESSENCE?

## 2. Method

### 2.1. Design and Participants

This study was part of a population-based study investigating the prevalence of Reactive Attachment Disorder (RAD) in 6–8 years old children from a sector of a UK city characterised by high levels of deprivation. For a detailed description of the methodology, see [11]. The prevalence paper predicted 23 RAD cases, made up of 13 children who were given a definite diagnosis of RAD and an additional 10 cases that would have been expected to have been diagnosed with RAD using an imputation dataset. 

This current paper describes the characteristics of the 13 children who were given a definite diagnosis of RAD and an additional 9 with a suspected or likely diagnosis of Reactive Attachment Disorder after screening of the total population of 1600 children. Of this sample (*n* = 22), fourteen completed the whole procedure; however, the remaining 8 children did not complete the cognitive or attachment measures. One family had moved away, one was uncontactable, and six opted out including three who felt the child's difficulties were too extreme to cope with the assessment, or they were already seeing enough professionals that they did not want to place additional burden on the child. 

### 2.2. Measures

#### 2.2.1. Strengths and Difficulties Questionnaire (SDQ)

The SDQ is a brief behavioural screening questionnaire for 3–16 years olds [12]. It contains 25 items, covering 5 subscales: emotional symptoms; conducts problems; hyperactivity/inattention; peer relationship problems; and prosocial behaviour. It can be completed by the children themselves, the caregiver, or the teacher. In this study, both the parent/carer and the teacher completed the SDQ.

#### 2.2.2. Relationship Problems Questionnaire (RPQ)

The RPQ is a 10-item parent and teacher-report screening instrument for RAD symptoms [13]. In a large general population twin sample, the RPQ had good internal consistency (Cronbach's alpha .85), and factor analysis identified that 6 items describe inhibited RAD behaviours and 4 items describe disinhibited RAD behaviours [13].

#### 2.2.3. Waiting Room Observation (WRO)

The waiting room observation is a structured observation of child behaviour with strangers in an unfamiliar waiting room setting [14]. It has been shown to discriminate between children with RAD and those without [14] as it identifies key relationship behaviours, for example, over friendliness with strangers. 

#### 2.2.4. Development and Well-Being Assessment (DAWBA)

The DAWBA is a screening questionnaire for a number of psychiatric diagnoses including emotional, behaviour, and hyperactivity disorders used with parents of children aged 2–17 years [15]. The DAWBA can be completed either using a paper format or, as in this study, using a computerised format. The parent is asked a number of closed questions, for example, “does he ever worry?” which, depending on the answer, may lead to a section being skipped or to more questions, for example, about how often the child worries. The DAWBA has been shown to be a valid measure of child psychopathology [15] and has been used in nationwide surveys of child and adolescent mental health [16].

#### 2.2.5. The Child and Adolescent Psychiatric Assessment, Reactive Attachment Disorder Module (CAPA-RAD)

The CAPA-RAD is a 28-item semistructured parent-report interview, which assesses RAD symptoms and is a module of the Child and Adolescent Psychiatric Assessment, a well validated semistructured parent-report interview for child psychopathology used in large epidemiological studies (CAPA) [17]. The CAPA-RAD has good interrater reliability with good discrimination [3].

#### 2.2.6. Social Skills Improvement System (SSIS)

The SSIS assesses social skills, problem behaviours, and academic competence and has been shown to have good reliability and validity [18]. In this 140-item questionnaire, the child's caregiver rates the frequency that their child displays various behaviours. 

#### 2.2.7. Manchester Child Attachment Story Task (MCAST)

The Manchester Child Attachment Story Task (MCAST) is a doll-play story stem technique measuring attachment patterns in middle childhood [19]. It includes four stories with attachment related themes using a dolls house, designed for use with school aged children. The child's story is videotaped and subjected to structured coding based on the SSP and Adult Attachment Interview (AAI) codes to provide an attachment classification [19]. It has good interrater reliability, stability of attachment patterns over time, and concurrent validity with well-validated measures of attachment [20].

#### 2.2.8. Wechsler Intelligence Scale for Children (WISC IV)

 The WISC is a scale of intelligence producing both a cognitive score (IQ) as well as scaled scores by age [21]. It can be used with children aged between 6 years and 16 years. It covers 4 domains: verbal comprehension; perceptual reasoning; working memory, and processing speed, with a full-scale IQ produced when these are combined. Extensive reliability and validity evidence was provided by Wechsler [21] and by Prifitera, Saklofske, and Weiss [22].

### 2.3. Procedure

Our results describe the characteristics of a group of children identified in an epidemiology study examining the prevalence of RAD. That study involved a 3-stage approach with 1,600 participants. The procedure of that study is described in detail in Minnis et al. [11]. In brief, the first stage involved parents and teachers both completing the SDQ and the RPQ. The second stage involved the parents completing the DAWBA, the CAPA-RAD, and the SSIS, while the third stage involved the child being assessed using the MCAST and the WISC-IV. All the data was reviewed, and where criteria for RAD was met, a diagnosis was made. This paper describes the characteristics of those children with a suspected or likely diagnosis of RAD.

#### 2.3.1. RAD Diagnoses

RAD diagnoses were made, based on DSM IV criteria, by HM and the research team, following review of the CAPA-RAD, the teacher RPQ, the Observational Checklist, 10 comorbid diagnoses (from the DAWBA), and videotaped interaction between the child and researcher (who was a stranger to the child at the assessment visit). In previous research, this has been shown to be highly sensitive and specific in discriminating children with RAD from typically developing children [3]. The child was given a “borderline/suspected” diagnosis when the diagnosis was not absolutely clear or when we were unable to see the child in school and were relying simply on interview and questionnaire data. Both DSM IV and ICD-10 suggest that RAD should only be diagnosed in the presence of a history of “pathogenic care.” It was decided that it would be upsetting for participants, and it would reduce response rates if we asked parents from the general population direct questions about abuse and neglect of their child, although this was explored to some extent in the posttraumatic stress disorder section of the DAWBA.

## 3. Results

We describe the characteristics of all 22 children with RAD behaviours. We gave 13 a definite diagnosis with the remaining 9 given a suspected or borderline diagnosis.

### 3.1. Demographics

We found that, of the 22 children with RAD behaviours, 13 (59.1%) were male and 9 (40.1%) were female. Ten (45%) of the children were thought to be living with birth parents, while 9 (41%) were known to be in foster care, and a further 3 (14%) were known to be in kinship care, living with a relative. 

### 3.2. Social Skills

Ten of the children (45.5%) were below average in the SSIS, as compared to American norms (UK norms are unavailable for the SSIS), while only 1 child scored above average in this measure. 

### 3.3. Attachment

**Figure 1:**
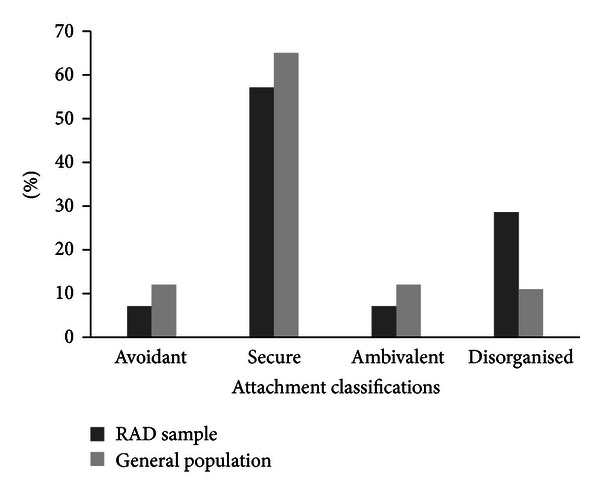
MCAST classifications in sample of RAD cases compared to the general population.

Attachment patterns of 14 of the children were classified using the MCAST and compared to general population norms. Of the 14 children included, 8 (57.1%) were given a secure attachment and 6 (42.9%) insecure. This is illustrated below and compared to the distribution which would be expected in a normative sample (Figure 1).

### 3.4. Problem Behaviours

**Figure 2:**
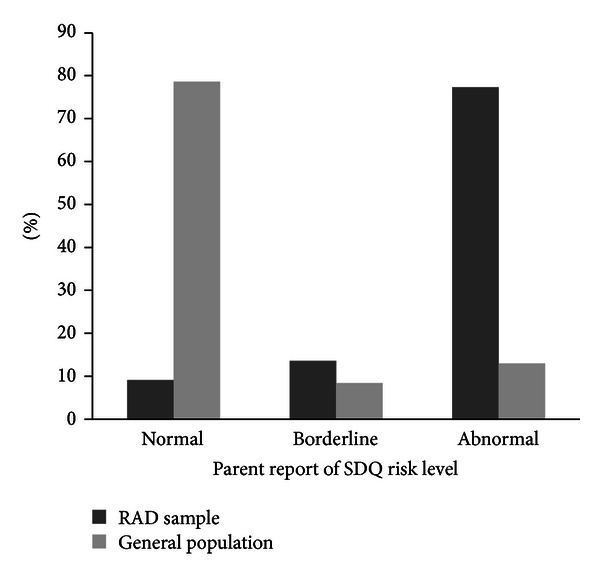
SDQ risk level for problem behaviours in sample of RAD cases compared to the general population.

The SDQ gives the total difficulties score which can characterise a child's risk of developing problems. Figure 2 shows the risk level of problem behaviours in the RAD sample, as reported by parents, and compares it with the risk level of the entire school sample from which this data was from, which is in line with the UK norms. 

### 3.5. Cognitive Ability

The WISC showed that the children in this sample were below average (100) in every aspect of this test of intellectual functioning (Table 1).

### 3.6. Psychiatric Diagnoses

The DAWBA is a screening tool for a number of psychiatric diagnoses based on ICD-10 and DSM IV criteria. The results showed that 11 (52%) had a likely diagnosis of attention deficit hyperactivity disorder (ADHD); 6 (29%) oppositional defiant disorder; 6 (29%) conduct disorder; 4 (19%) posttraumatic stress disorder (PTSD); 3 (14%) an autism spectrum disorder (ASD); 3 (14.3%) a specific phobia; and 1 (5%) a tic disorder. Overall, over 85% of the children identified as having RAD in this sample had another diagnosis predicted by the DAWBA. 

All but one of the children with a definite diagnosis of RAD had histories of definite or suspected maltreatment documented during the DAWBA interviews with parents or carers, and all but two of those with a borderline/suspected diagnosis of RAD had such a history. In the others, a history of maltreatment was impossible to determine but may well have been present; we made a child protection referral regarding one child on whom there was no clear previous history of maltreatment.

## 4. Discussion

We described the characteristics of 22 children with a suspected, borderline, or definite diagnosis of RAD. We found that they have a high level of comorbidity with other disorders, had lower IQs than population norms, had a higher level of disorganised attachment than has been found in general population studies, more problem behaviours, and had lower social skills than would be found in the general population. These findings are in line with previous research about children coming from institutions. This study shows that those children in the general population with RAD also have these additional problems, providing further evidence that a multidisciplinary approach is needed when working with these children, in line with the research on ESSENCE.

We found that over half of our sample had a secure attachment; this offers support to the growing body of research showing that RAD and insecure attachment are not the same thing [3]. We did, however, also find that there was a higher rate of disorganised attachment than would be found in the general population. This is not surprising as research has previously shown that those with a history of maltreatment have a greater chance of having a disorganised attachment in later development. 

### 4.1. Implications of Results

#### 4.1.1. Clinical Implications

The results of this study demonstrate that reactive attachment disorders are present in the general population. Previous research has shown that this may be as a result of both environmental factors and genetics [13]. Children who begin their lives with compromised/disrupted attachment are at significant risk for subsequent developmental difficulties including low self-esteem, lack of emotional regulation, difficulty with peer/social relationships, lack of empathy, and behavioural difficulties, any number and combination of which may see the child present to specialist children's services. 

This has implications for the assessment, intervention, and education of this group of children when they present with difficulties. Aspects of the RAD presentation such as indiscriminate friendliness (a core feature of disinhibited reactive attachment disorder) may be overlooked in a child who presents from the general population. This has potential implications for targeting the most appropriate and effective intervention for the child. Such presenting difficulties should not be underestimated when there is a suggestion of a history of pathogenic care. 

This study also demonstrates the high levels of comorbidity with other disorders including ADHD. There are potential implications from a formulation and intervention perspective if there is a lack of awareness of RAD presenting within the general population group leading to exclusive treatment of the “comorbid” disorder coupled with a lack of recognition of the child's difficulties in forming and maintaining relationships.

#### 4.1.2. In the Classroom

Most teachers in recent years have become more aware of the diverse needs of children in the classroom, and ongoing professional development will include training in attachment issues, autism, ADHD, and other social/emotional difficulties [23, 24]. The problem for the class teacher may be that children who have RAD may not be easily identifiable and are therefore not considered to be in an “at risk” group. So, while the teacher may have some kind of classification system to help support the children with specific conditions such as autism or ADHD, there is a need to raise awareness that there are some children who may well suffer from more than one psychiatric condition or educational difficulty. Support plans need to be flexible and individually tailored for each child with a difficulty.

Within a class situation, misbehaviour can escalate to exclusion for a child. For children who have been traumatised or abused, it is particularly important that they feel a part of the class and the wider school community with inclusion being even more vital for neglected or abused children [25]. Success for these children is essential for their emotional wellbeing, and training on good techniques to support vulnerable children has to involve all school staff members, not just teachers [26].

This study used the WISC to measure the cognitive ability of the children in this sample. Despite being a measure of general intelligence, there are components which are indicative of school taught material. We suspect that, due to both early maltreatment and behaviour issues, children with RAD may have been more likely to miss out on educational opportunities. This suggests that with proper learning support, cognitive scores could improve for the children in our sample.

### 4.2. Limitations

The findings here describe the characteristics of only a small sample of children; however, they are the first children with RAD to be described from a total population. In addition, these children were compared to population norms as opposed to a matched control group. 

## 5. Conclusions

Our findings demonstrate that, even when identified through population screening, children with RAD have a complex presentation that fits well within the ESSENCE group of disorders. The logical conclusion to be drawn from this is that children presenting with RAD symptoms will require a detailed holistic assessment looking for comorbid disorders, cognitive, and language problems.

## Figures and Tables

**Table 1:** Average scores in the WISC in our sample of children with RAD (*n* = 14).

	Mean (SD)	Range
Verbal IQ	82 (14.3)	63–119
Perceptual reasoning	83.7 (11.9)	65–104
Working memory	82.9 (11.5)	62–104
Processing speed	87.1 (10.5)	68–106
Full-scale IQ	79.36 (12.0)	56–106
